# Identification determinant factors on willingness to pay for health services in Iran

**DOI:** 10.1186/s13561-017-0179-x

**Published:** 2017-11-21

**Authors:** Javad Javan-Noughabi, Zahra Kavosi, Ahmad Faramarzi, Mohammad Khammarnia

**Affiliations:** 1 Health Management and Economics Research Center, Iran University of Medical Sciences, Tehran, Iran; 2 Department of Health Economics, School of Health Management and Information Sciences, Iran University of Medical Sciences, Tehran, Iran; 3 Health Human Resources Research Center, Shiraz University of Medical Sciences, Shiraz, Iran; 4 Department of Health Management and Economics, School of Public Health, Tehran University of Medical Sciences, Tehran, Iran; 5 Health Promotion Research Center, Zahedan University of Medical Sciences, Zahedan, Iran

**Keywords:** Willingness to pay, Altruistic, Private, Iran

## Abstract

**Background:**

A common method used to examine the relationship between internal preferences and caring externalities is willingness to pay (WTP) approach. We aimed to estimate WTP for health status with different severity level and identify determinant factors on WTP.

**Methods:**

For determining main factors in WTP, a cross-sectional study was conducted in Shiraz in the southeast of Iran, in March to April 2015. The open-ended method was used to estimate monthly WTP in private and altruistic section. Multivariate regression analyses using ordinary least squares were applied to examine the effect of Scio–demographic factors on WTP using SPSS software 21.

**Results:**

Participants were willing to pay an average amount of $ 295 in health status 1 and an average amount of $ 596 in health status 6 (worst status) for internal preferences. Altruistic WTP for health status 1 was $ 294 and participants were willing to pay an average amount of $ 416 in health status 6. Multiple regression analysis identified monthly income as the key determinant of WTP for internal preferences and caring externalities (*P* < 0.01). With an increase of 1% in income, private WTP increase 1.38% in health status 1.

**Conclusions:**

The finding indicates that the mean of WTP increases at severe health status; therefore, health policy maker should allocate resources toward severe health status.

## Background

Many economists believe that healthcare is different in ways that generate market failure, therefore it is important for formulating public policy in the health sector [1–3]. externalities is one of the most discussed in health market failure, an external effect is existed when benefits and costs of an activity by some agent accrue to someone not directly involved in the activity, and this effect is not prized by the market [4].

In the economic evaluation are existed some procedures for measuring of caring externalities that one approach is altruistic willingness to pay (WTP). Auguste Comte first time used the concept of altruism and believed that there are two separate forces or motivation in every human, one of them focused on their own interests that are selfishness and other force focused on others and the interests of others that is altruism [5].

WTP is the maximum amount of income an individual is willing to give up to ensure that a proposed service or good is available [6].

In the health sector, various studies have been estimated WTP with different methods for health services. For instance, Wang et al. (2015) done study on the Impacts of Healthy Eating and Anti-Obesity Advertising on Willingness-to-Pay by Consumer Body Mass Index [7, 8]. X Yu et al. (2014) estimated WTP for the “Green Food” in China [9] Dror found that using bidding game among 3024 households at the rural location in India, about two-third of sample agree to WTP for health insurance [10]. Basu (2013) applied contingent valuation approach to estimate willingness to pay for prevent Alzheimer’s disease and demonstrated the mean of WTP is $155 per month [11].

Open – ended is an approach which is used for the measurement of WTP, in this approach as respondent is asked, “How much would you be willing to pay to be cured?” [12] this approach has been frequently used in the economic evaluation [13–17]. For example, Li et al. (2017) used open-ended format to estimate to WTP for newborn screening test for spinal muscular atrophy which their study showed that People expressed a willingness to pay for spinal muscular atrophy screening even without an available therapy (median: $142; mean: $253). Willingness to pay increased with treatment availability and respondent income [18, 19].

The relationship between caring externalities and internal preferences (altruistic WTP and private WTP) for health status with different severity levels and determinant factors on WTP were studied as the purpose of the current research. The results of the study could be helpful for health policy makers and mangers in accurate planning in health system.

## Methods

### Setting and sample

A cross-sectional study was conducted among adults that have income from the general population in Shiraz, Iran, in March to April 2015. Shiraz is the center of health care and medical tourism in Iran and is located in the southeast of Iran [20]. The design of the study was explorative; therefore, it was important to obtain the views of different social groups. By that, the participants were selected from different settings. The sample size was determined using the following equation in which *p* = 0.8, and d = 0.055 [21].

$$ N=\frac{Z_{1-{\frac{a}{2}}^{p\left(1-p\right)}}}{d^2}=200 $$

For sampling, the population was divided into 9 areas then on average 25 participants was randomly selected of each region.

The used scenarios in the study was based on that participants do not have insurance to pay for different status or services are not free, in addition health status were independent from each other. Data collection was conducted by open-ended questionnaire in a face-to-face interview. To recognize of external factors that might influence on WTP in this study, the participants were asked to provide their sex, educational background, employment status, and income per month. In other parts of the questionnaire, it was possible to compare private WTP and altruistic WTP.

### Data collection tool

Data was collected by questionnaire, which had included two parts; the first part was included demographic variable (sex, educational background, employment status and income per month). In other part, the questionnaire asked from participants to declare private WTP and altruistic WTP in six level of health status. Six levels regarding mobility(physical activity) were used from a scale constructed by Nord [22]; which included: first, can move about without difficulty anywhere, but has difficulties with walking more than a kilometre. Second, can move about with difficulty at home, but has difficulties in stairs and outdoors. Third, moves about with difficulty at home, needs assistance in stairs and outdoors. Fourth, can sit, needs assistance to move about - both at home and outdoors. Fifth, to some degree bedridden can sit on a chair in part of the day if helped up by others and sixth, completely bedridden.

### WTP measurement

There are four ways to measure WTP in economic evaluation: open-ended, take-it-or-leave-it (or alternatively discrete choice), payment card, and bidding games types of questions [23, 24]. We chose the open-ended method for this study, this method has previously been used in many studies, in addition there is little information for altruistic and private willingness to pay in health care and the open-ended technique is a good method for obtaining first estimates [25, 26].

We applied private WTP and altruistic WTP for evaluation internal preferences and caring externalities. To estimate private WTP, the question dealt with the health status of participants and was asked as respondent in current way: “If you are suffering from the different health status. How much are you willing to pay to be cured from each health state?”

The scenarios were used to estimate the altruistic WTP the same as in private WTP, but the question relates to others health status and was asked as participants in following way: “suppose a stranger person suffers as described health status and you don’t know exactly who she/he is, but he/she cannot be treated due to inability to pay medical expenses. How much are you willing to pay for her/him treatment of any health status?”

### Data analysis

Data was analysed on STATA 13 version. The data analysis was started with descriptive analysis (frequency and mean) which would allow us to explore the data and identify specific trend of the study’s variables. In the descriptive phase of the study, private WTP compared to altruistic WTP. In addition, since data in private WTP had non-normal distribution; therefore, logarithm was used for analysis while there was no need to get Logs for altruistic WTP data. Moreover, to test the effect of explanatory variables on private WTP and altruistic WTP was applied of OLS regression in each health status.

The below model was performed in analysis:

$$ {\mathrm{Y}}_{\mathrm{i}}=\upalpha +{\upbeta}_1{\mathrm{X}}_{1\mathrm{i}}+{\upbeta}_2{\mathrm{X}}_{2\mathrm{i}}+.\dots +{\mathrm{e}}_{\mathrm{i}}\kern3.25em \mathrm{i}=1\dots \dots .\mathrm{n} $$

Where Y_i_ denotes natural logged private WTP; however, for altruistic WTP is dollar ($), α is a constant, X_i_ denotes the control variables, β represents the coefficient and e is an error term.

- Ethics approval and consent to participate:

This study was approved by Ethic Committee of Shiraz University of Medical Sciences.

## Results

### Scio-demographic characteristics of the study population

A total of 200 participants take part in this study. More than 60% of them have academics education. Furthermore, more than of 50% of participants were male. (See Table 1).

**Table 1:** The average monthly income of participants regarding to demographics variables

Variables		Frequency (%)	Month mean (USA $)	*P* value
Sex	Male	111 (55.5)	866	0.001
	Female	89 (44.5)	508.5	
Age	<35	112 (56.0)	667	0.112
	≥35	88 (44.0)	759	
Marital Status	Single	93 (46.5)	748	0.201
	Married	107 (53.5)	670.5	
Education	non-academics	66 (33.0)	519.5	0.001
	academics	134 (67.0)	799.5	

According to the results, about 88.8% of the participants were willing to pay for health status. The study results showed that the mean of participants’ income was US$ 707. They were willing to pay an average amount of $ 295 in health status 1 and an average amount of $ 596 in health status 6 (worst status) for internal preferences. Moreover, altruistic WTP for health status 1 was $ 294 and participants were willing to pay an average amount of $ 416 in health status 6.

According to Table 1, the mean of monthly income was significant for gender and education (*P* = 0.001 and *P* = 0.001, respectively.). So male participants had higher income rather than females and persons with academic education had higher income rather than others. Table 1 shows monthly income for participant regarding to demographic variables.

**Fig. 1:**
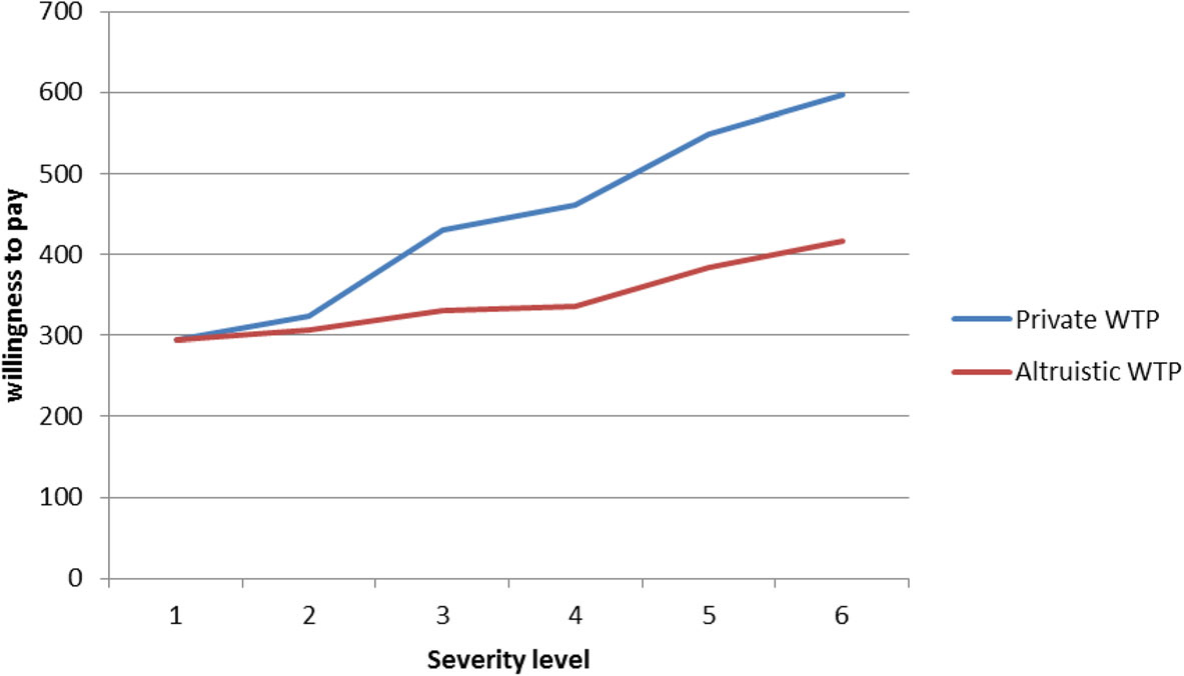
The mean of private and altruistic WTP in the southwest of Iran

Private WTP compare to altruistic WTP for different health status are shown in Fig. 1. Based on the Figure, the mean value of private WTP is higher than the mean value of the altruistic WTP in all health status and difference in the WTP is higher in the sever status. This means that, the mean value of private WTP for owns improvement from first and last health status were $ 295 and $ 596 respectively. But, Altruistic WTP for others health improvement from first and sixth health status were $ 294 and $ 416 respectively. This demonstrates that the respondents had understood the different scenarios.

Table 2 shows determinant factors on private WTP in different status of health. For example, model 1 shows the effect of studied variables on the private WTP for first health status that explanted in the method. Regression analysis showed that among the studied variables only monthly income of participant had significantly influenced on private WTP in all different status of health in significant level of 0.01. For instance, in first health status, the increase of 1% of the monthly income results in 1.3% increase in the private WTP. Moreover, in significant level of 0.01, sex had significant effect on private WTP for first and second health status (Model one and two).According to Table 2, in significant level of 0.05, only education status had significant effect on private WTP for fourth health status (Model four).

**Table 2:** Ordinary least square regression for private WTP (Log) in different health status

Variables	Model 1	Model 2	Model 3	Model 4	Model 5	Model 6
	WTP (SE)	WTP (SE)	WTP (SE)	WTP (SE)	WTP (SE)	WTP (SE)
Cons	−8.07^b^	−5.85^b^	−2.59^b^	−1.94^b^	−1.31^b^	−.53
	(0.82)	(0.75)	(0.56)	(0.65)	(0.40)	(0.45)
Sex (ref = male)	0.21^b^	0.16^b^	0.07	0.07	0.01	−0.03
	(0.06)	(0.05)	(0.04)	(0.04)	(0.03)	(0.03)
Age (ref = less than 35 year)	0.04	0.01	−0.007	0.01	−0.03	0.01
	(0.06)	(0.05)	(0.04)	(0.04)	(0.03)	(0.03)
Marital status (ref = single)	0.10	0.09	0.02	0.02	0.004	−.03
	(0.06)	(0.05)	(0.04)	(0.04)	(0.03)	(0.03)
Education (ref = nonacademic)	0.02	0.02	0.05	0.11^a^	0.04	−0.02
	(0.06)	(0.05)	(0.04)	(0.05)	(0.03)	(0.03)
Income	1.38^b^	1.27^b^	1.1^b^	1.06^b^	1.05^b^	1.02^b^
	(0.04)	(0.04)	(0.03)	(0.03)	(0.02)	(0.02)
R square	0.82	0.83	0.87	0.82	0.92	0.90

^a^Significant level at 0.05

^b^Significant level at 0.01

Table 3 shows determinants factors that effect on the person’s WTP who tendency to pay for other’s health improvement (altruistic WTP). Regression analysis in Table 3 showed that monthly income of respondents significantly influenced on altruistic WTP in all different status of health (*P* < 0.01). For example, in first health status (Model one), the increase of 1% of the monthly income results in $ 251 increase in the altruistic WTP. Also, in significant level of 0.01, sex in model two, three and four had statistically significant on altruistic WTP. Moreover, in significant level of 0.05, sex had significant effect on altruistic WTP for first and fifth health status (Model one and five).

**Table 3:** Ordinary least square regression for altruistic WTP in different health status

	Model 1	Model 2	Model 3	Model 4	Model 4	Model 4
	WTP (SE)	WTP (SE)	WTP (SE)	WTP (SE)	WTP (SE)	WTP (SE)
Cons	−1330.26^b^	−1274.28^b^	-1311^b^	−1245.95^b^	−1341.48^b^	−1314.88^b^
	(115.69)	(112.04)	(106.55)	(111.79)	(210.38)	(337.53)
Sex (ref = male)	−48.12^a^	−58.22^b^	−58.38^b^	−60.29^b^	−85.53^a^	−102.45
	(19.71)	(19.09)	(18.15)	(19.04)	(35.84)	(57.5)
Age (ref = less than 35 year)	27.08	25.9	33.17	26.81	−23.99	1.36
	(20.06)	(19.43)	(18.48)	(19.39)	(36.49)	(58.54)
Marital status (ref = single)	35.17	32.9	28.31	25.98	67.98	−15.53
	(19.49)	(18.88)	(17.95)	(18.83)	(35.45)	(56.87)
Education (ref = nonacademic)	1.93	3.7	4.97	2.91	16.95	23.13
	(20.24)	(19.6)	(18.64)	(19.56)	(36.81)	(59.06)
Income	251.1^b^	246.76^b^	255.65^b^	249.30^b^	274.76^b^	292.54^b^
	(15.41)	(14.92)	(14.19)	(14.89)	(28.02)	(44.95)
R square	0.65	0.66	0.7	0.67	0.42	0.24

^a^Significant level at 0.05

^b^Significant level at 0.01

## Discussion

The study was designed to determine important factors which are effective on private and altruistic WTP in Iran. We found that a large proportion of participants had willing to pay for one’s own (private WTP) and for others’ (altruistic WTP) hypothetical health improvement. Moreover, amount of private WTP and altruistic WTP increases with worsening health status; however, in all of health status the value of private WTP was higher than value of altruistic WTP and the difference becomes further in the severity health status. A similar study conducted by Jacobsson in Sweden found that the mean value of private WTP and altruistic WTP for sixth health status were $1000 and $8000 respectively, which were higher than from our study [27]. The difference of WTP in our study compared to Jacobsson could be several reasons. First, the study was conducted in countries that are different as culturally, so the willingness to pay is usually lower in developing countries, for example, the average of WTP for health insurance in the USA in 2008 was 75 to $ 125; however, this amount was $ 47 in Namibia [28, 29]. Second, Jacobsson’s study was conducted in 2001, so it could be discounted the time- value of money. According to the formula of the discount rate, the value of money has been decreased over time [30, 31].

Our results have indicated income is an important predictor for willingness to pay. If income increases 1%, private WTP will be increased 1.38% and 1.02% in health status 1 and 6, respectively. Moreover, with an increase of 1% of income, the altruistic WTP is increased $ 251 and $ 292 in health status 1 and 6, respectively. In line with the study’s findings, Ahmed et al. 2015, Wright et al. 2009, Krupnick et al. 2002, also observed significant association between WTP and income [28, 32, 33].

Our analysis was explained that none of demographic variables has significant relationship with WTP for different health status however, gender, for example in the health status 1 the value of private WTP for women is higher than men but the amount of altruistic WTP for men is higher. There are no similar previous studies which confirm our results.

There are some limitations of the present study that need to be considered. One potential limitation of open–ended methods is related to bias that participants express the value of WTP incorrectly. Second, it is possible that respondent don’t understand which scenarios are independent from each other, so WTP for each strategy was related to other scenarios. Third, it is difficult to disclose the real income of the participants; in particular the study was designed at the individual level and cross sectional.

## Conclusions

This current study is provided evidence on WTP for health status and demonstrated that a large proportion of participants had WTP for health status. The value of WTP was difference for internal preference and caring externalities. This study indicates that the mean of WTP increases at severe health status, therefore health policy maker should allocate resources toward severe health status. Among the Scio-economics and demographic factors only income and gender was associated with the WTP significantly.
